# Multiple primary malignancies involving primary sporadic colorectal cancer in Japan: incidence of gastric cancer with colorectal cancer patients may be higher than previously recognized

**DOI:** 10.1186/s12957-014-0432-2

**Published:** 2015-02-07

**Authors:** Takaharu Kato, Koichi Suzuki, Yuta Muto, Junichi Sasaki, Shingo Tsujinaka, Yutaka J Kawamura, Hiroshi Noda, Hisanaga Horie, Fumio Konishi, Toshiki Rikiyama

**Affiliations:** Department of Surgery, Saitama Medical Center, Jichi Medical University, 1-847 Amanuma-cho, Omiya-ku, Saitama 330-8503 Japan; Tsudanuma Central General Hospital, 1-9-17 Yatsu, Narashino-shi, Chiba 275-0026 Japan; Nerima Hikarigaoka Hospital, 2-11-1 Hikarigaoka, Nerima-ku, Tokyo 179-0072 Japan

**Keywords:** Colorectal cancer, Gastric cancer, Multiple primary malignancies

## Abstract

**Background:**

Improvement in the prognosis of colorectal cancer (CRC) patients has led to increasing occurrences of multiple primary malignancies (MPMs) alongside CRC but little is known about their characteristics. This study was undertaken to clarify the clinical and pathological features of MPMs, especially those at extra colonic sites, in patients with CRC.

**Methods:**

We reviewed 1,111 patients who underwent operations for primary sporadic CRC in Saitama Medical Center, Jichi Medical University between April 2007 and March 2012. Two patients with familial adenomatous polyposis, one with hereditary non-polyposis colorectal cancer, two with colitic cancer, and any patients with metastasis from CRC were excluded. We compared the clinicopathological features of CRC patients with and without MPMs.

As a control, we used a database compiled of patients with gastric cancer (GC) detected by mass screening performed in the Saitama Prefecture in Japan 2010 and compared these with CRC patients with synchronous GC.

**Results:**

Multiple primary malignancies at extracolonic sites were identified in 117 of 1,111 CRC patients (10.5%). The median age was 68 (range, 29 to 96) versus 71 (50 to 92) (*P* < 0.001). The incidence of GC (44.4% (52 of 117)) was the highest of all MPMs. All CRC patients with GC were older than 57 years. Synchronous GC was detected in 26 patients.

By contrast, out of 200,007 screened people, 225 people were diagnosed as having GC in the Saitama Prefecture. The age-standardized incidence of synchronous GC in CRC patients was significantly higher (0.53%) than in the control group (0.03%) (odds ratio, 18.8; 95% confidence interval, 18.6 to 19.0; *P* < 0.001).

**Conclusion:**

Patients with CRC who were older than 50 years preferentially developed GC synchronously and metachronously. Thus, this patient group should undergo careful perioperative screening for GC.

## Background

Colorectal cancer (CRC) is a common form of cancer in developed countries, being the second and third leading cause of cancer death in men and women, respectively [1], and has become an important issue in Asian countries [2,3]. Reports from the World Health Organization show that the incidence of CRC is rapidly rising in Asian countries [2,4,5], which have experienced a two- to four-fold increase in the incidence of CRC during the last few decades [6]. Patients with CRC also frequently develop multiple primary malignancies (MPMs) [7,8]. The prognosis for CRC patients has steadily improved, owing to both increased detection of early CRC and development of new chemotherapeutic agents. The improvement in the prognosis for CRC patients has led to an increased incidence of MPMs [9]. It is important to understand the characteristics of MPMs, in order to administer appropriate treatment and to determine suitable follow-up plans in CRC patients. This study was undertaken to review the clinical and pathological features of MPMs, especially those of extracolonic sites, in CRC patients.

## Methods

Between April 2007 and March 2012, 1,111 patients underwent surgery for primary sporadic CRC in Saitama Medical Center, Jichi Medical University. From our database of CRC patients, we extracted details of patients who had developed at least one MPM in an extracolonic site while having synchronous or metachronous CRC. Synchronous MPMs were defined as malignancies detected within 6-month interval before and after the detection of CRC [9,10].

All the MPMs were pathologically or cytologically confirmed as malignancies. Any metastasis to extracolonic sites from the CRC was excluded.

Two patients with familiar adenomatous polyposis, one with hereditary non-polyposis colorectal cancer, and two with ulcerative colitis associated colorectal cancer were also excluded. The tumor locations of CRC were divided into two groups: colon and rectum (portions below the level of the sacral promontory) [11]. We reviewed clinicopathological parameters, such as age, sex, location of tumor, differentiation of tumor, stage according to the tumor, node, and metastasis (TNM) classification, of the American Joint Committee on Cancer (6th edition) [12], and occurrence of MPMs at extracolonic sites.

It is not known whether the incidence of gastric cancer (GC) is higher or not in CRC patients. We performed gastroscopy for CRC patients as screening because the incidence of GC is high in Japan [13]; if GC was discovered in CRC patients before surgery, we could treat them by simultaneous operation. Of 1,111 patients, 832 underwent gastroscopy. These features in patients with CRC harboring simultaneous GC were compared with those in patients with GC that was detected by mass screening. Screening for GC is widely spread in Japan, a nation afflicted by high GC mortality [14]. In 1983, a national law was enacted that stipulated that each municipality should provide annual GC screening for inhabitants aged 40 or older, by either gastroscopy or barium meal. In 2010, 200,007 residents (6.0% of the target population) participated in this mass screening in the Saitama Prefecture and GC was detected in 225 patients (0.01%).

### Statistical analysis

Clinical and pathological factors related to the presence of MPMs were compared by the Mann-Whitney *U* test and the *χ*^2^ test. To enable the incidence of synchronous GC in patients with CRC to be compared, allowing for the differences in age distributions between patients with CRC and people who underwent screening for stomach abnormalities, as described, an age-adjusted odds ratio was calculated, comparing the rate in patients with synchronous GC with that in patients whose GC was diagnosed after screening. Statistical significance was indicated for *P* < 0.05.

The odds ratio was presented with 95% confidence intervals. SPSS version 17 (IBM, Tokyo, Japan) was used for statistical analyses.

In this study retrospective anonymized clinical information from patients from the Saitama Medical Center, Jichi Medical University were employed. The study has been approved by the Research Ethics Committee at Jichi Medical University.

## Results and discussion

Among the 1,111 patients with primary CRC, there were 117 patients (10.5%) with MPMs. Table 1 shows the clinicopathological relationship between CRC patients with and without MPMs. Patients with CRC and MPMs were older than patients without MPMs (*P* < 0.001). All patients with CRC and MPMs were 50 years old or older.

**Table 1:** **Comparison of clinical and pathological features between colorectal cancer patients with and without multiple primary malignancies**

	**Colorectal cancer without multiple primary malignancies**	**Colorectal cancer with multiple primary malignancies**	***P*** ^**a**^
	**(** ***n*** **= 994: 89.5%)**	**(** ***n*** **= 117: 10.5%)**	
Age (years):^b^	68 (29 to 96)	71 (50 to 92)	<0.001^c^
<50	79 (100)	0 (0)	0.002
≥50	915 (88.7)	117 (11.3)	
Sex:			0.26
Male	627 (88.7)	80 (11.3)	
Female	367 (90.8)	37 (9.2)	
Tumor location:			0.345
Colon	778 (89.0)	96 (11.0)	
Rectum	216 (91.1)	21 (8.9)	
Differentiation:			0.678
Well or moderate	981 (89.4)	116 (10.6)	
Poor	13 (92.9)	1 (7.1)	
TNM stage:			0.036
I	247 (87.0)	37 (13.0)	
II	333 (87.6)	47 (12.4)	
III	295 (91.9)	26 (8.1)	
IV	119 (94.4)	7 (5.6)	

^a^ *χ* ^2^ test, except as marked; ^b^median values (range); ^c^Mann-Whitney *U* test.

Values in parentheses are percentages unless indicated otherwise.

**Figure 1:**
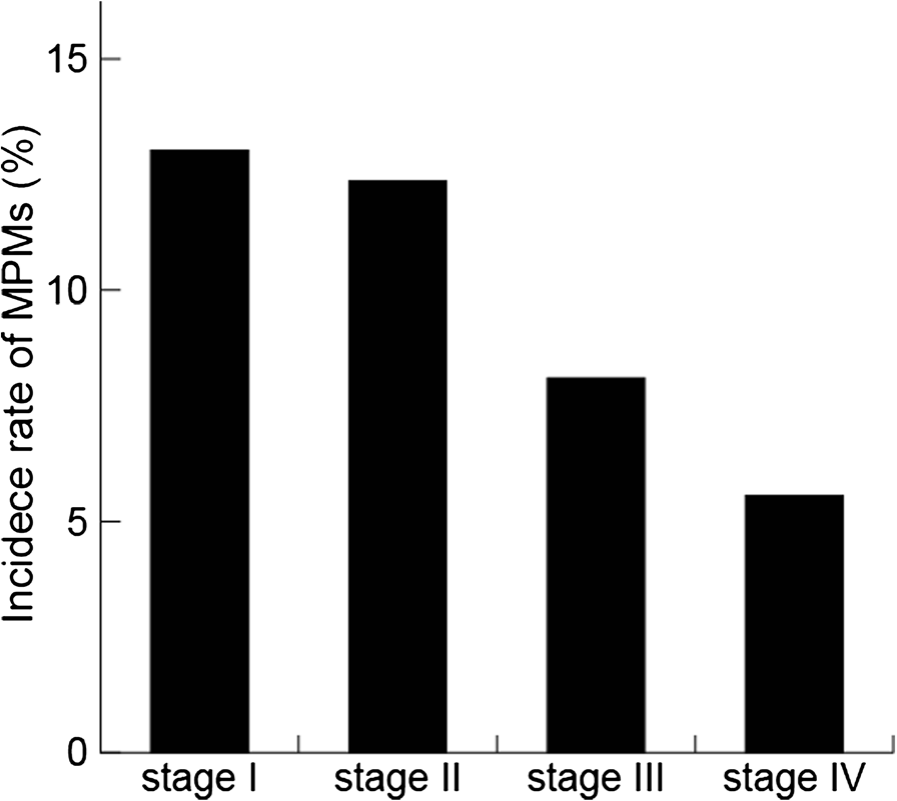
**Incidence of multiple primary malignancies according to colorectal cancer stage.** 37 of 284 patients with colorectal cancer (13%) with stage I had multiple primary malignancies, 47 of 330 patients with colorectal cancer (12.4%) had stage II, 26 of 321 patients with colorectal cancer (8.1%) had stage III and 7 of 126 patients with colorectal cancer (5.6%) had stage IV. The incidence of multiple primary malignancies decreased with colorectal cancer stage. MPM, multiple primary malignancy.

There were no statistical differences between sexes, tumor location, or differentiation of tumor. Early CRC tended to be more associated with MPMs than advanced CRC (*P* = 0.034) (Table 1 and Figure 1). Nine patients had triple primary malignancies.

Table 2 shows the sites of the MPMs. Of 18 organs noted to have MPMs in this study, malignancies in the stomach were most frequently associated with CRC, both synchronously and metachronously, followed by lung or breast cancer.

**Table 2:** **Extracolonic site distribution of multiple primary malignancies in patients with colorectal cancer**

**Organ**	**Total**	**Percentage**	**Number of synchronous patients with multiple primary malignancies**	**Number of metachronous patients with multiple primary malignancies**
Stomach	52	39.4	26	26
Lung	17	12.9	7	10
Breast	10	7.58	0	10
Kidney	8	6.06	2	6
Esophagus	7	5.3	5	2
Prostate	7	5.3	2	5
Bladder	5	3.79	1	4
Thyroid	4	3.03	0	4
Hematologic	4	3.03	1	3
Uterus	3	2.27	1	2
Liver	2	1.52	1	1
Bile duct	2	1.52	1	1
Ovary	2	1.52	0	2
Tongue	1	0.76	0	1
Laryngeal	1	0.76	1	0
Anal fistula	1	0.76	1	0
Penis	1	0.76	0	1
Skin	1	0.76	0	1
Total	128	100	49	79

**Figure 2:**
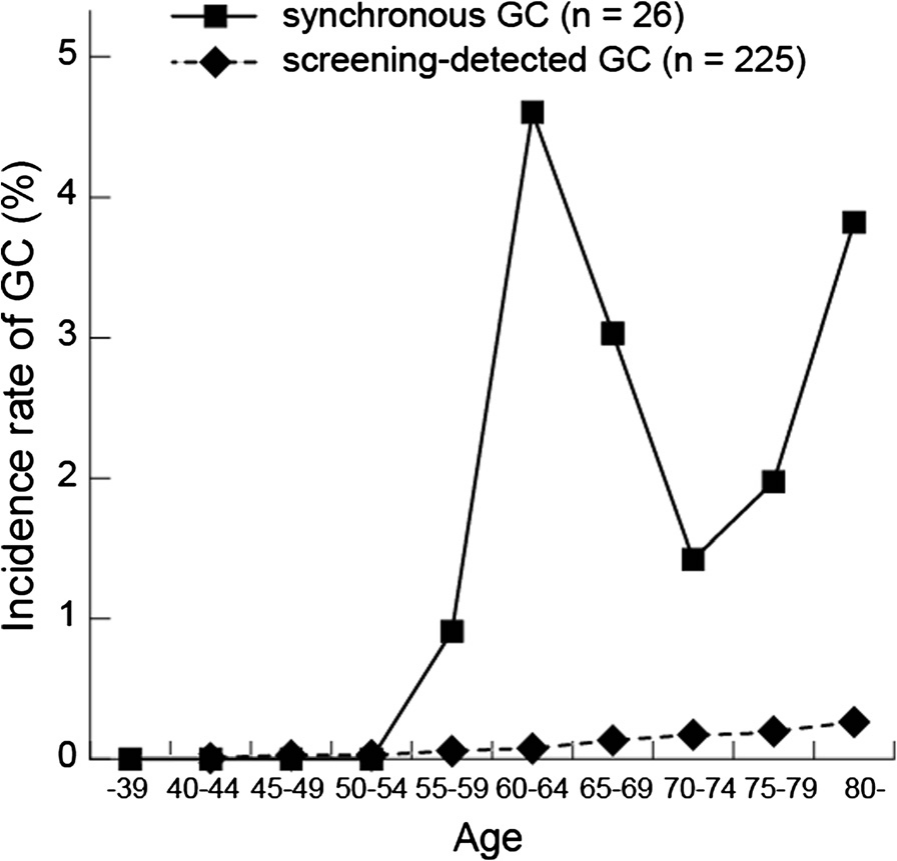
**Comparison of the incidence between synchronous gastric cancer in patients with colorectal cancer and gastric cancer among screened patients according to age subgroups.** In those older than 54 years, the incidence of synchronous gastric cancer in patients with colorectal cancer was significantly higher than that of gastric cancer among the mass-screened group. GC, gastric cancer.

**Figure 3:**
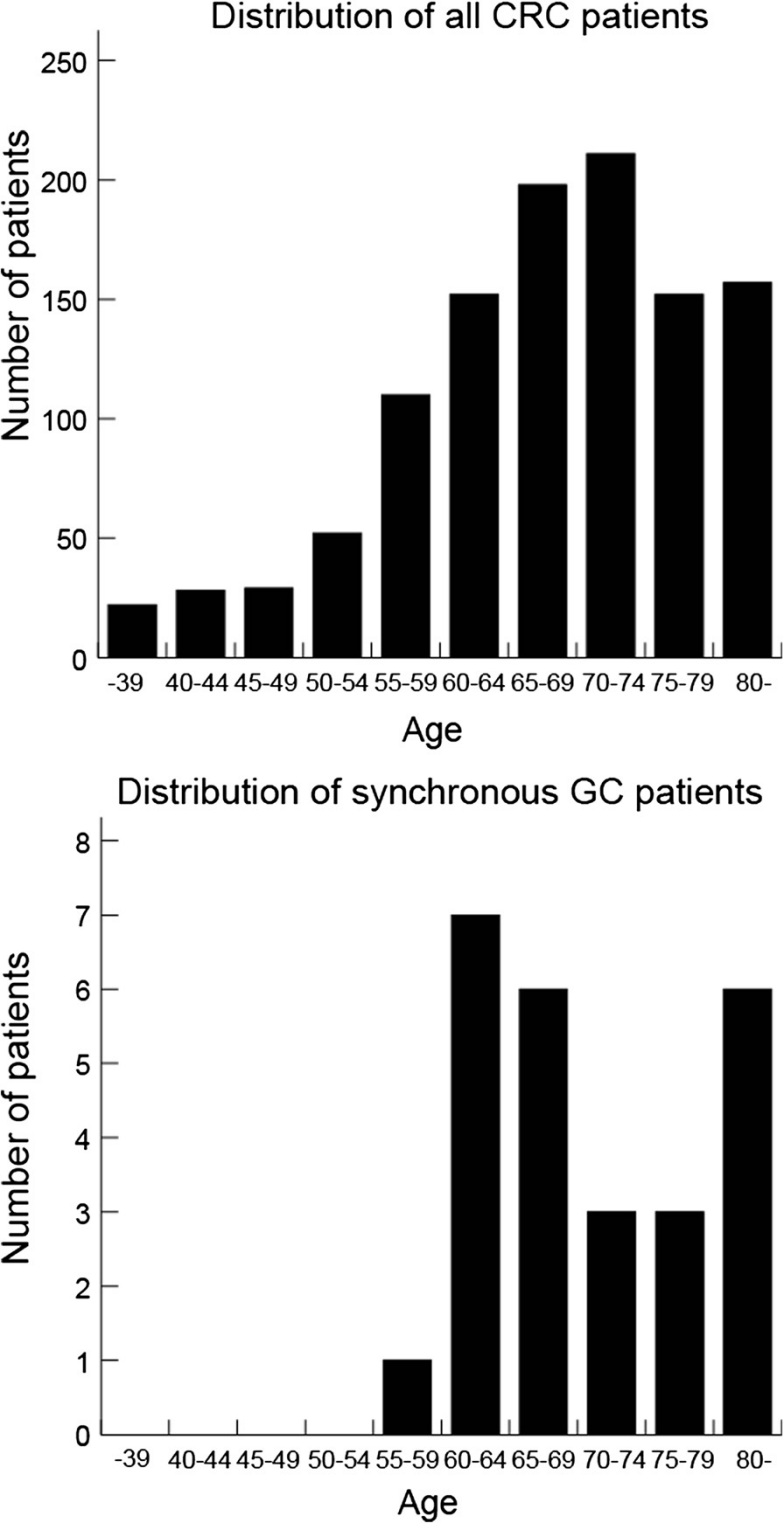
**Comparison of the age distributions between all patients with colorectal cancer** **(top panel)** **and patients with colorectal cancer with synchronous gastric cancer** **(bottom panel).** The age distribution of all patients with colorectal cancer peaked in the 70 to 74 years age range (211 of 1,111 patients). By contrast, that of patients with colorectal cancer with synchronous gastric cancer formed two peaks; among individuals 60 to 64 years old (7 of 152 patients) and among those 80 years or older (6 of 157 patients). CRC, colorectal cancer; GC, gastric cancer.

Patients with CRC and synchronous GCs were compared with GC patients detected by mass screening carried out in the Saitama Prefecture in 2010 (Tables 3 and 4). The incidence of synchronous GC in patients with CRC was significantly higher than that of GC in the mass-screened group: 2.34% versus 0.11% (Table 3 and Figure 2). The age-adjusted odds ratio for incidence of GC in the mass-screened group was 18.8 (95% confidence interval, 18.6 to 19; *P* < 0.001) (Tables 3 and 4 and Figure 2). All patients with CRC with synchronous GC were 55 years old or older. The age distribution of all CRC patients peaked in the 70s. Conversely, that of CRC patients with synchronous GCs formed two peaks; one for individuals in their 60s and one for individuals who were 80 years old or older (Figure 3).

**Table 3:** **Comparison of clinical features between synchronous gastric cancer with colorectal cancer patients and gastric cancer among screened group**

	**Colorectal cancer patients with synchronous gastric cancer**	**Gastric cancer among screened group**	***P*** ^**a**^
	**(** ***n*** **= 26/1,111)**	**(** ***n*** **= 225/200,007)**	
Age (years)	69 (58–87)	71 (42–95)	0.600^b^
Sex:			0.66
Male	20 (76.9)	164 (72.9)	
Female	6 (23.1)	61 (27.1)	
Incidence	2.34	0.11	<0.001^c^

^a^ *χ* ^2^ test, except as marked; ^b^Mann-Whitney *U* test; ^c^age-adjusted.

**Table 4:** **Comparison of the incidence of gastric cancer between colorectal cancer patients and screened patients**

	**Synchronous gastric cancer patients**		**Gastric cancer patients among screened group**		**Odds ratio for gastric cancer among screened group**
**Age (years)**	**Gastric cancer**	**All colorectal cancer**	**Gastric cancer**	**All screened people**	
<40	0	22	0	4,138	Invalid value
40 to 44	0	28	1	12,659	Invalid value
45 to 49	0	29	3	11,515	Invalid value
50 to 54	0	52	3	11,729	Invalid value
55 to 59	1	110	9	16,087	16.37 (2.06, 130.48)
60 to 64	7	152	26	34,902	64.76 (27.67, 151.57)
65 to 69	6	198	56	42,917	23.92 (10.18, 56.17)
70 to 74	3	211	62	35,615	8.41 (2.62, 2701)
75 to 79	3	152	80	20,515	10.31 (3.15, 33.68)
≥80	6	157	26	9,930	15.14 (6.14, 37.31)
All ages	26	1,111	225	200,007	18.8 (18.6 to 19.0)^a^

^a^age-adjusted.

Values in parentheses are 95% confidence intervals.

This study demonstrates that patients with CRC are likely to develop MPMs in extracolonic sites, the occurrence of which accounted for 10.5% (117/ 1,111) in our analyses. This result is congruent with previous reports showing the occurrence of MPMs in extracolonic sites among CRC patients, which ranged from 2.4% to 17% [14-17]. Moreover, our data suggest that patients with early-stage CRC are more likely to exhibit MPMs than those with advanced stages of CRC; this might be because the prognosis in early CRC patients is favorable, and thus the follow-up time becomes longer than that for advanced CRC patients. Therefore, the MPMs might occur more often in patients with early-stage CRC than in those with advanced CRC.

Our data showed that GC was the most frequently associated cancer in patients with CRC both synchronously and metachronously, consistent with previous reports [9,10,15-19].

Note that increased surveillance for GC might appear to increase its incidence as a result of increased detection. Patients with CRC do seem to undergo far more testing than the general population, including gastroscopy and computed tomography, accounting for the high detection rate of GC in patients with CRC [20]. As we have mentioned before, annual GC screening for inhabitants was undertaken not only by gastroscopy but also by barium meal. In some areas of the Saitama Prefecture, individuals can choose between barium meal and gastroscopy for the screening of GC. Out of 200,007 screened people, 56,036 people (28.0%) underwent gastroscopy in 2010. We should agree on the difficulty of comparison between the incidence of synchronous GC and that of screen-detected GC. In addition, 2 of 52 patients with GC were diagnosed with stomach cancer first. After that, the two patients were diagnosed with CRC by screening colonoscopy. So, the present study has some selection bias, increasing the incidence of GC with CRC. Consequently, it is hard to determine precisely the real magnitude of the correlation between CRC and GC. There are few reports of MPMs in CRC patients with comparative data [10,17], and even then, the existing data were calculated from a population that included unscreened people; hence, it is far from accurate. After calculation of prevalence using populations that consist of screened subjects, we should employ this as comparative data. Allowing for this point, we used a database of patients with GC that was detected by screening carried out in the Saitama Prefecture in Japan in 2010 as comparative data, to highlight a characteristic of synchronous GC with CRC. Some researchers reported that screend groups were likely to differ from unscreened groups in certain characteristics, such as familial history, diet, or smoking habits [21]. Though we could not dispute this bias, we viewed the GC data from screened individuals as a preferable comparison because the population included many unscreened people. From these results, the prevalence of synchronous GC in patients with CRC (2.6%) was found to be extremely high, which implies that patients with CRC are at increased risk of synchronous GC. In addition, all patients with CRC who had synchronous GC were older than 50 years. Based on these data, careful perioperative screening is necessary for the detection of GC in patients with sporadic CRC who are older than 50 years.

In addition to the finding that the most common site of a metachronous MPM in CRC patients is the stomach [22], one of the most common MPMs in patients with GC is CRC [23,24]. These facts seem to suggest that GC and CRC share the same risk factors. In a previous study, microsatellite instability [19], translocation of TP53 [25,26] and ageing [22,27,28] were regarded as shared risk factors between GC and CRC. In terms of ageing, the median age of CRC patients with MPMs was significantly higher than that of CRC patients without MPMs, which agrees with those reports. Intriguingly, the age distribution of CRC patients with synchronous GC formed two characteristic peaks; not only was there a peak at 80 years old or older, but there was also a peak for people in their 60s. Familiar adenomatous polyposis or hereditary non-polyposis colorectal cancer are given as hereditary diseases to be complicated with GC and CRC. We excluded patients with these diseases from our cohort of patients with CRC by checking patients’ familial histories. Additionally, in hereditary non-polyposis colorectal cancer, the mean age at diagnosis as CRC is 42 to 50.4 years [29,30]. In familiar adenomatous polyposis, without surgical intervention, patients almost inevitably develop CRC by a mean age of 40 or 50 years [31]. We concluded that these hereditary diseases were less likely to be included. It is thought that there are some factors that are not due to ageing or known hereditary disease.

In the 18 organs that had MPMs, the lung was the second most frequently associated with CRC, followed by the stomach, and then the breast. In Japan, the organ with the highest incidence of MPMs in Japan is the stomach, followed by the colon and rectum, and then the lung and breast [13]. This sequence matches precisely with that of MPMs in our cohort of patients with CRC.

There are some limitations to this study. First, this research was performed retrospectively. Second, our data did not include information regarding *Helicobacter pylori* infection status. It is believed that 70 to 80% of people over 40 years of age in Japan are infected with *H. pylori* [32]. *H. pylori* infection is an important factor that has been associated with the development of GC [33]. The incidence of *H. pylori* infection of the stomach in European countries is less than that in Japan [34]. In a European study of MPMs with CRC, cancer in the stomach is far from frequent [35]. In this study, *H. pylori* might contribute to the high incidence of GC with CRC.

Finally, our data did not include any information regarding atrophic gastritis; a previous study revealed that atrophic gastritis might be a major cause of GC [36]. For these reasons, additional studies are required for further evaluation.

## Conclusions

Gastric cancer was the most common synchronous or metachronous MPM in patients with CRC. Careful perioperative screening is necessary for the detection of MPMs in patients with CRC. However, screening for GC is not always necessary in sporadic CRC patients younger than 50 years.

## Abbreviations

CRC: colorectal cancer
GC: gastric cancer
MPM: multiple primary malignancy
TNM: tumor, node, and metastasis
